# A meta-analysis of risk factors for depression in adults and children after natural disasters

**DOI:** 10.1186/1471-2458-14-623

**Published:** 2014-06-19

**Authors:** Bihan Tang, Xu Liu, Yuan Liu, Chen Xue, Lulu Zhang

**Affiliations:** 1 Institute of Military Health Management, Second Military Medical University, 800 Xiangyin Rd, Shanghai 200433, China

**Keywords:** Depression, Risk factors, Children, Adults, Natural disasters

## Abstract

**Background:**

A number of studies have shown a range of negative psychological symptoms (e.g. depression) after exposure to natural disasters. The aim of this study was to determine risk factors for depression in both children and adults who have survived natural disasters.

**Methods:**

Four electronic databases (PubMed, Embase, Web of Science, and PsychInfo) were used to search for observational studies (case–control, cross-sectional, and cohort studies) about depression following natural disasters. The literature search, study selection, and data extraction were conducted independently by two authors. Thirty-one articles were included in the study, of which twenty included adult participants and eleven included child participants. Summary estimates were obtained using random-effects models. Subgroup analysis, sensitivity analysis, and publication bias tests were performed on the data.

**Results:**

The prevalence of depression after natural disasters ranged from 5.8% to 54.0% in adults and from 7.5% to 44.8% in children. We found a number of risk factors for depression after exposure to natural disasters. For adults, the significant predictors were being female ;not married;holding religious beliefs; having poor education; prior trauma; experiencing fear, injury, or bereavement during the disaster; or losing employment or property, suffering house damage as a result of the disaster. For children, the significant predictors were prior trauma; being trapped during the disaster; experiencing injury, fear, or bereavement during the disaster; witnessing injury/death during the disaster; or having poor social support.

**Conclusions:**

The current analysis provides evidence of risk factors for depression in survivors of natural disasters. Further research is necessary to design interventions to improve the mental health of survivors of natural disasters.

## Background

Natural disasters have a devastating impact on affected regions and their populations, often causing death and serious personal injury. Survivors of the disaster often experience mental health problems in the aftermath. One of the most common mental health problems for survivors of natural disasters is depression [1]. Depression is a risk factor for a range of diseases and poor health outcomes [2]. It is important to understand what factors may give rise to depression following a natural disaster. Studies investigating the prevalence of depression after such events indicate that the percentage of people that experience depression ranges from 4.9% to 54% [3-33]. Such variability can only be partially explained by the differences among these studies in diagnostic tools, sampling frames, and study design [20]. Differences in basic characteristics, trauma characteristics, and post-trauma characteristics may explain the large variation in depression rates between studies.

The onset of depression following natural disasters has been studied for more than 20 years. Studies have demonstrated that sometimes there is a delay in the onset of depression in both children and adults. Depression can be experienced weeks or months after the natural disaster, and in some cases persists for years [34,35]. Several meta-analyses have been carried out on risk factors for depression in different populations. These have focused on populations affected by humanitarian emergencies, such as refugees, internally displaced persons, populations affected by mass conflict/displacement, and populations affected by deadly diseases such as cancer and stroke [36-39]. To date, however, there has been no meta-analysis of risk factors for depression in populations specifically affected by natural disasters.

Understanding risk factors for experiencing depression after natural disasters can help clinicians provide more tailored treatments to reduce symptoms and aid post-disaster recovery. This study investigates the determinants of depression in survivors of natural disasters using a systematic meta-analysis of observational studies.

## Methods

Methods and reporting were in accordance with MOOSE (meta-analysis of observation studies in epidemiology) guidelines (The Additional file 1) [40].

### Data sources and search strategy

Observational studies (case–control, cross-sectional, and cohort studies) on risk factors for depression after natural disasters published in English were included in our meta-analysis, irrespective of publication status and article type. Two investigators (B. T. and X. L.) conducted a systematic literature search using the electronic databases PubMed (from 1965 to April 2014), Embase (from 1965 to April 2014), Web of Science (from 1986 to April 2014) and PsychInfo (from 1990 to June 2013). As describing in Additional file 2: Table S1, we used MeSH terms (“mental disorders”, “Depression”, “Depressive Disorder” , “Earthquakes” , “Tsunamis” , “Floods” , “Cyclonic Storms” , “Volcanic Eruptions” , “Tornadoes” , “Landslides” , “Droughts”) and free texts for the PubMed search, Emtree (“depression”, “mental disease”, “earthquake” , “tsunami” , “flooding” , “'hurricane” , “landslide” , “drought” , “natural disaster”) for the Embase search, and free texts for Web of Science and PsycINFO search. Additionally, manual searches of references cited in all relevant original and review articles were conducted. If there were some full texts unavailable in the databases, we attempted to obtain information from the authors by email.

### Selection and exclusion criteria

In order for studies to be eligible for inclusion in the meta-analysis, they had to fulfil the following criteria: (1) was an epidemiological investigation of risk factors for depression after natural disasters; (2) reported the relative risks (RRs) or odds ratios (ORs) and corresponding 95% confidence intervals (CIs) for risk factors in the development of depression; (3) included risk factors for depression after the natural disasters which we studied; and (4) included a study sample of children, adults, or both. The exclusion criteria were as follows: (1) used depression score as a variable, but did not obtain ORs or RRs; (2) were not published in English; and (3) focused on participant groups known to be susceptible to depression (i.e. pregnant women and people suffering from mental illness). Studies in which most of the study sample was less than 18 years old were classified as child studies; otherwise, they were classified as adult studies. If more than one article reported data from the same population, then the most recent and complete article was included in our meta-analysis. Study selection and application of inclusion criteria were carried out independently by the two investigators who conducted the literature search (B.T. and X.L.).

### Data extraction and quality assessment

Data extraction was independently performed by two investigators (B.T. and Y.L.). The following information was extracted from each eligible study: first author’s surname, year of publication, study location, disaster type, study design, study population, diagnosis of depression, sample size, depression prevalence, interval between research and the date of the natural disaster, age and gender of participants, estimated effect size (OR/RR), corresponding 95% CI, and covariates adjusted in the statistical analysis. For studies that reported several multivariable-adjusted effect estimates, we selected the one that adjusted for more potential confounding variables.

Quality assessments were conducted independently by two investigators (B.T. and X.L.) using an 11-item instrument recommended by the Agency for Healthcare Research and Quality (AHRQ) for cross-sectional studies [41] and the 9-star Newcastle-Ottawa Scale (NOS) [42] for case–control and cohort studies. Studies that recorded a score of seven stars or more were considered high quality. The quality assessment of the original articles was re-examined and adjudicated independently by an additional investigator (L.Z.).

### Search results and characteristics of studies

The Additional file 3: Figure S1 shows the complete selection process. Up to April 2014, 5,967 records were retrieved by our search strategy. We excluded 5,788 articles after reading the titles and abstracts, and retained 179 articles for further evaluation by reading the full texts. The Additional file 4: Table S2 shows the 148 excluded articles and detailed reasons for exclusion after full-text reading. Finally, we selected 31 full-text articles about risk factors for depression after natural disasters for our meta-analysis [3-33]. There were 21 articles that focused on earthquakes, 7 on hurricanes/tornadoes/typhoons, 2 on tsunamis, and 1 on floods. Twenty studies investigated the association between risk factors and depression in adult survivors of natural disasters, totalling 4,548 depression cases out of 28,217 participants. Eleven studies investigated the association between risk factors and depression in child survivors of natural disasters, totalling 2,816 depression cases out of 12,890 participants. Table 1 shows the general characteristics of the 31 studies included in the analysis.

**Table 1:** General characteristic of the included studies with regard to risk factors for depression after natural disasters

**Id**	**Author**	**Year**	**Country**	**Disaster type**	**Study design**	**Population**	**Diagnosis of depression**	**Sample size**	**Depression prevalence**	**Interval between research and disaster**	**Male%**	**Age**	**Quality**
1	Guo	2014	China	Wenchuan earthquake	cross section	adults	Center for Epidemiologic Studies Depression Scale	633	22.9%	6 months	41.7%	31-86	7
2	Nillni	2013	Armenia	Hurricane Katrina	cross section	adults	Patient Health Questionnaire-9	810	11.7%	18 month	47.8%	> = 18	6
3	Boscarino	2013	US	Hurricane Sandy	cross section	adults	A major depressive disorder scale	200	6.0%	6 months	35%	> = 18	6
4	Zhou	2013	China	Wenchuan earthquake	cross section	adults	Structured Clinical Interview for DSM-IV-TR axis I disorder	14207	11.0%	6 months	48.42%	>15	6
5	Gigantesco	2013	Italy	L’Aquila earthquake	cross section	adults	Patient Health Questionnaire 8	895	5.8%	14–19 months	49.2%	18-69	7
6	Cheng	2013	China	Wenchuan earthquake	cross section	adults	Structured Clinical Interview for DSM-IV Axis I Disorders	182	48.9%	12 months	34.8%	> = 18	6
7	Cerda	2013	Haiti	Haiti earthquake	cross section	adults	Patient Health Questionnaire 9	1315	28.3%	2–4 months	28.9%	> = 18	6
8	Vu	2012	Armenia	Hurricane Katrina	cohort	adults	18-item Vietnamese depression scale	128	8.5%	12 month	66.4%	28–52	7
9	Zhang	2012	China	Yushu earthquake	cross section	adults	Hopkins Symptoms Checklist-25	505	38.6%	3–4 months	53.5%	16 - 87	7
10	Zhang	2012	China	Wenchuan earthquake	cross section	the Elderly	Hopkins Symptoms Checklist‒25	274	35.2%	14 months	38%	60-98	9
11	Zhang	2011	China	Wenchuan earthquake	cross section	adults	Hopkins Symptoms Checklist-25	1181	49.6%	1 year	37.3	16-98	9
12	Tracy	2011	America	Hurricane Ike	cross section	adults	Patient Health Questionnaire-9	658	4.9%	2-5 months	49.4%	> = 18	7
13	Paranjothy	2011	England	UK floods	cross section	adults	Patient Health Questionnaire-9	2113	13.3%	3 months and 6 months	48%	16-96	6
14	Anwar	2011	Pakistan	Pakistan earthquake	cross section	women	Hopkins Symptom Checklist-25	387	54.0%	4 years	-	15-49	7
15	Amstadter	2009	Vietnam	typhoon Xangsane	cross section	adults	Structured Clinical Interview for DSM-IV	798	5.9%	3 months	-	18-96	6
16	Van Griensven	2006	Thailand	Thailand Tsunami	cross section	adults	Hopkins Symptom Checklist-25	1061	20.4%	1 month	38.6%	15-90	6
17	Chou	2007	China (taiwan)	Chi-Chi earthquake	cross section	adults	Mini-international Neuropsychiatric Interview	216	11.6%	6 month, 2 years, 3 years	45.8%	> = 16	6
18	Acierno	2007	France	Florida hurricane	cross section	adults	Structured Clinical Interview for DSM-IV	1452	6.1%	6-9 months	48.1%	> = 18	6
19	Chou	2005	Taiwan (china)	Chi-Chi earthquake	cross section	adults	Mini-international Neuropsychiatric Interview	442	9.5%	4-6 months	48.4%	> = 16	7
20	Armenian	2002	Armenia	Armenian Earthquak	case control	adults	Structured Clinical Interview for DSM-III-R	760	52.0%	2 year	45.1%	13-70	9
21	Adams	2014	Armenia	Spring 2011 tornado	cross section	children	National Survey of Adolescents Depression module for DSM-IV	1514	7.5%	8.8 months	49.1%	12-17	8
22	Ye	2014	China	Wenchuan earthquake	cross section	children	Depression Self-rating Scale for Children	1573	27.4%	6 months	45.8%	15 (mean)	7
23	Pan	2013	China	Wenchuan earthquake	cross section	children	Zung self-rating Dep11ression Scale	362	44.8%	3 years	43.6%	11-16	8
24	Kadak	2013	Turkey	Van earthquake	cross section	children	Child Depression Inventory	738	37.7%	6 months	55.0%	13-17	6
25	Wang	2012	China	Wenchuan earthquake	cross section	children	Depression Self-rating Scale for Children	1841	19.5%	10 months	48.7%	11-20	7
26	Liu	2011	China	Wenchuan earthquake	cross section	children	Trauma Symptom Checklist for Children	330	14.5%	6 months and 12 months	50.0%	8-12	4
27	Fan	2011	China	Wenchuan earthquake	cross section	children	The Depression Self-rating Scale for Children	2081	24.5%	6 months	45.9%	14.5 (mean)	6
28	Lau	2010	China	Wenchuan earthquake	cross section	children	Children’s Depression Inventory	3324	22.6%	1 month	54.3%	12-18	7
29	Jia	2010	China	Wenchuan earthquake	cross section	children	Children’s Depression Inventory	596	13.9%	15 months	49.8%	8 - 16	7
30	Thienkrua	2006	Thailand	Thailand tsunami	cross section	children	Birleson Depression Self-Rating Scale	371	8.4%	2 months and 9 months	46.9%	7-14	6
31	Eksi	2007	Turkey	Turkey earthquake	cross section	children	Structured Clinical Interview for DSM-IV	160	30.6%	6-20 weeks	36.3%	9-18	6

### Classification of risk factors

According to previously published studies [14,43,44], risk factors for depression among children and adults after natural disasters were divided into three categories: basic characteristics (including age, gender, education, marital status, religious beliefs, prior trauma and prior physical illness), trauma characteristics (including being trapped; experiencing fear, injury, or bereavement, e.g. losing close friends or family members; or witnessing injury/death as a result of the natural disasters), and post-trauma characteristics (including amount of social support, employment, loss of property, and house damage).

### Statistical analysis

We examined risk factors for depression after natural disasters by looking at the adjusted ORs and 95% CIs reported in each study. A random-effects model [45], which assumes that the true underlying effect varies among included studies, was used to estimate the pooled RRs with 95% CIs. Heterogeneity between studies was evaluated by the χ^2^ test and I2 statistic [46]. The probability of publication bias was assessed with the Egger’s regression test [47]. Subgroup analyses and sensitivity analyses were performed after excluding low-quality studies, unadjusted results, research on the Wenchuan earthquake, and studies long after the disaster onset (>6 months). Twelve research articles on the Wenchuan earthquake, which made up nearly half (38.7%) of the incorporated articles, were excluded to explore whether this would make a significant change to the results. We also excluded studies that were implemented a long time after the onset of the disaster (>6 months). Having a long period between the research and the natural disaster raises the possibility that participants may have been exposed to subsequent traumatic events in addition to the natural disaster, which may confound the interpretation of results. If risk factors were multi-categorical variables, we used an OR of the highest versus lowest category (such as age in some studies, education level, scare, house damage, loss of property, and social support). If risk factors were continuous variables (such as age in some studies), they were excluded to avoid inaccuracy because it is not appropriate to combine the ORs from continuous and segmental data.

Stata Version 12.0 software (Stata Corp, College Station, TX) was used for all analyses and all statistical tests were two-sided. A value of p < 0.05 was considered an indication of statistical significance.

## Results

### Risk factors for depression in adults

**Figure 1:**
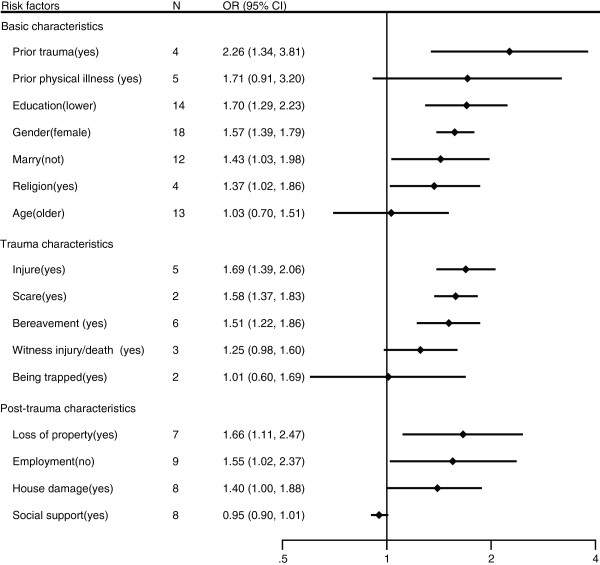
Risk factors for depression after natural disasters in adults.

The prevalence of depression in adults after natural disasters ranged from 5.8% to 54.0%. The risk factors for depression after natural disasters in adults are presented in Table 2 and Figure 1. Regarding the basic characteristics of survivors, we found that being female, having a low-level education, not being married, following a religion, and prior trauma were significantly associated with depression after natural disasters, with pooled ORs of 1.57 (95% CI, 1.39–1.79), 1.70 (95% CI, 1.29–2.23), 1.43 (95% CI, 1.03–1.98), 1.37 (95% CI, 1.02–1.86), and 2.26 (95% CI, 1.34–3.81), respectively. However, heterogeneity was found for education (I2 = 72.7%, p < 0.001), marriage (I2 = 73.7%, p < 0.001), prior trauma (I2 = 67.9%, p = 0.025), and prior physical illness (I2 = 81.3%, p < 0.001). The subgroup and sensitivity analyses showed inconsistencies in the results for marriage, religion, prior trauma, and prior physical illness, which should be interpreted with caution because of potential bias. In addition, we found a publication bias for education (Egger’s test p = 0.008) and marriage (Egger’s test p = 0.030). Thus, after adjusting for publication bias, the OR was 1.31 (95% CI, 0.98–1.76) for education and 1.43 (95% CI, 1.03–1.98) for marriage.

**Table 2:** Risk Factors for depression after natural disasters in adults

	**All studies**					**High quality**		**Adjustment**		**Exclude wenchuan**		**Within 6 months**	
	N	OR (95% Cl)	I^2^ (P value)	Egger test	Trim and fill	N	OR (95% Cl)	N	OR (95% Cl)	N	OR (95% Cl)	N	OR (95% Cl)
**Basic characteristics**													
Age (older)	13	1.03 (0.70-1.51)	80.8%	P = 0.839	-	5	1.37 (0.86-2.17)	8	0.98 (0.56-1.70)	8	0.93 (0.61-1.44)	6	0.81 (0.37-1.78)
			(p < 0.001)										
Gender (female)	18	1.57 (1.39-1.79)	18.5%	p = 0.282	-	8	1.60 (1.35-1.90)	13	1.65 (1.43-1.89)	13	1.61 (1.41-1.84)	10	1.74 (1.50-2.02)
			(p = 0.233)										
Education (lower)	14	1.70 (1.29-2.23)	72.7%	P = 0.008	1.31 (0.98-1.76)	7	1.44 (1.04-1.99)	7	1.93 (1.20-3.10)	9	1.48 (1.13-1.93)	9	2.18 (1.41-3.37)
			(p < 0.001)										
Marry (not)	12	1.43 (1.03-1.98)	73.7%	P = 0.030	1.43 (1.03-1.98)	5	1.15 (0.95-1.40)	6	2.47 (0.90-6.78)	7	1.02 (0.80-1.30)	7	1.47 (0.88-2.47)
			(p < 0.001)										
Religion (yes)	4	1.37 (1.02-1.86)	0%	P = 0.469	-	2	1.51 (1.07-2.13)	-	-	2	1.25 (0.74-2.11)	2	1.25 (0.74-2.11)
			(p = 0.635)										
Prior trauma (yes)	4	2.26 (1.34-3.81)	67.9%	P = 0.352	-	1	1.50 (0.80-2.60)	2	1.73 (1.33-2.25)	4	1.73 (1.33-2.25)	2	2.85 (0.90-8.96)
			(p = 0.025)										
Prior physical illness (yes)	5	1.71 (0.91-3.20)	81.3%	P = 0.175	-	2	1.34 (0.99-1.83)	2	2.42 (0.58-10.08)	1	4.28 (1.78-10.30)	2	1.59 (0.24-10.35)
			(p < 0.001)										
**Trauma characteristics**													
Being trapped (yes)	2	1.01 (0.60-1.69)	0%	-	-	-	-	1	0.94 (0.54-1.62)	1	0.94 (0.54-1.62)	2	1.01 (0.60-1.69)
			(p = 0.465)										
Scare (yes)	2	1.58 (1.37-1.83)	0%	-	-	2	1.58 (1.37-1.83)	2	1.58 (1.37-1.83)	-	-	-	-
			(p = 0.535)										
Injure (yes)	5	1.69 (1.39-2.06)	0%	P = 0.559	-	3	1.83 (1.43-2.35)	4	1.66 (1.36-2.04)	2	1.49 (1.12-1.97)	3	1.55 (1.19-2.02)
			(p = 0.655)										
Witness injury/death (yes)	3	1.25 (0.98-1.60)	0%	P = 0.120	-	2	1.32 (0.92-1.88)	-	-	2	1.32 (0.92-1.88)	2	1.23 (0.93-1.64)
			( p = 0.931)										
Bereavement (yes)	6	1.51 (1.22-1.86)	0%	P = 0.454	-	4	1.43 (1.11-1.83)	4	1.54 (1.21-1.97)	3	1.49 (1.12-1.99)	2	1.52 (1.12-2.05)
			(p = 0.906)										
**Post-trauma characteristics**													
Social support (yes)	8	0.95 (0.90-1.01)	90.1%	P = 0.014	0.95 (0.90-1.01)	4	0.99 (0.95-1.03)	6	0.95 (0.89-1.01)	5	0.5 (0.31-0.80)	2	0.92 (0.77-1.10)
			(p < 0.001)										
Employment (no)	9	1.55 (1.02-2.37)	89.4%	P = 0.703	-	5	1.57 (1.30-1.91)	9	1.55 (1.02-2.37)	6	1.80 (1.04-3.12)	4	1.85 (0.85-4.01)
			(p < 0.001)										
Loss of property (yes)	7	1.66 (1.11-2.47)	74.7%	P = 0.512	-	5	1.32 (0.92-1.91)	3	2.02 (0.92-4.43)	5	1.91 (1.15-3.20)	3	2.14 (1.01-4.54)
			(p = 0.001)										
House damage (yes)	8	1.40 (1.00-1.88)	51.3%	P = 0.308	-	5	1.65 (1.04-2.63)	3	1.66 (0.89-3.10)	4	1.38 (1.01-1.89)	6	1.37 (0.94-2.01)
			(p = 0.045)										

With regard to the trauma characteristics of survivors, people who experienced fear, injury, or bereavement during a natural disaster were more likely to suffer from depression, with pooled ORs of 1.58 (95% CI, 1.37–1.83), 1.69 (95% CI, 1.39–2.06), and 1.51 (95% CI, 1.22–1.86) with no heterogeneity or publication bias. All the results were consistent according to the subgroup and sensitivity analyses.

Finally, analysis of the post-trauma characteristics of survivors showed that unemployment (OR 1.55, 95% CI, 1.02–2.37), loss of property (OR 1.66, 95% CI, 1.11–2.47), and house damage (OR 1.40, 95% CI, 1.00-1.88) were related to depression. However, heterogeneity was found for unemployment (I2 = 89.4%, p < 0.001), loss of property (I2 = 74.7%, p = 0.001), and house damage (I2 = 51.3%, p < 0.045), suggesting that findings on these variables show inconsistencies.

### Risk factors for depression in children

**Figure 2:**
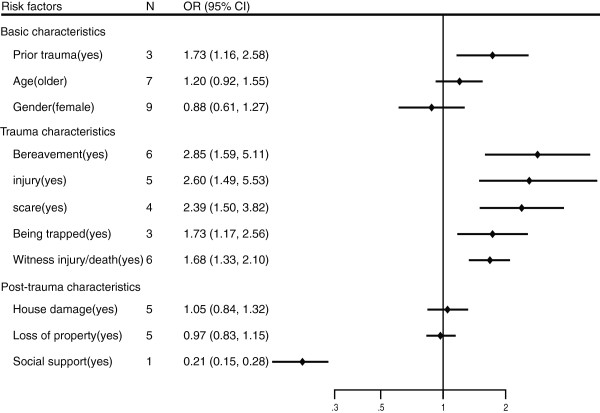
Risk factors for depression after natural disasters in children.

The prevalence of depression in children after natural disasters ranged from 7.5% to 44.8%. The risk factors for depression after natural disasters in children are presented in Table 3 and Figure 2. Regarding the basic characteristics of survivors, the pooled analysis had shown that only prior trauma was associated with risk of depression (OR 1.73 95% CI 1.16-2.58) with high heterogeneity (I2 = 86.6%, p = 0.001). However, after excluding the Wenchuan earthquake data, prior trauma was associated with the risk of depression, but this relationship was not significant. In addition, gender and age were not related to risk of depression.

**Table 3:** Risk factors for depression after natural disasters in children

	**All studies**					**High quality**		**Adjustment**		**Exclude wenchuan**		**Within 6 months**	
	N	OR (95%cl)	I^2^	Egger test	Trim and fill	N	OR (95%cl)	N	OR (95%cl)	N	OR (95%cl)	N	OR (95%cl)
			(P value)										
**Basic characteristics**													
Age (older)	7	1.20 (0.92-1.55)	73.5%	P = 0.663	-	2	1.01 (0.67-1.52)	6	1.60 (0.88-1.53)	3	1.10 (0.75-1.62)	4	1.21 (0.84-1.74)
			(p =0.001)										
Gender (female)	9	0.88 (0.61-1.27)	87.9%	P = 0.023	0.88 (0.61-1.27)	4	0.94 (0.50-1.76)	7	0.92 (0.61-1.39)	4	0.51 (0.26-0.99)	4	0.83 (0.44-1.56)
			(p <0.001)										
Prior trauma (yes)	3	1.73 (1.16-2.58)	86.6%	P = 0.555-	-	2	1.94 (1.21-3.11)	3	1.73 (1.16-2.58)	2	1.82 (0.95-3.49)	2	1.47 (1.21-1.78)
			(p = 0.001)										
**Trauma characteristics**													
Being trapped (yes)	3	1.73 (1.17-2.56)	0%	P = 0.218	-	1	1.70 (1.02-2.84)	2	2.15 (0.82- 5.65)	1	6.03 (0.79-45.93)	2	2.15 (0.71-6.47)
			(p = 0.464)										
Scare (yes)	4	2.39 (1.50-3.82)	52.9%	P = 0.110	-	2	2.20 (0.10-4.88)	2	2.20 (0.10-4.88)	1	2.73 (1.02-7.29)	1	3.07 (1.47-6.40)
			(p = 0.095)										
Injury (yes)	5	2.60 (1.49-5.53)	49.1% (p = 0.097)	P = 0.722	-	2	2.69 (0.69-10.44)	3	3.01 (1.17-7.72)	3	3.08 (1.23-7.73)	2	3.54 (1.99-6.30)
Witness injury/death (yes)	6	1.68 (1.33-2.10)	42.5%	P = 0.461	-	3	1.43 (1.00-2.04)	4	1.46 (1.09-1.94)	2	2.11 (1.15-3.86)	2	2.48 (1.46-4.21)
			(p = 0.122)										
Bereavement (yes)	6	2.85 (1.59-5.11)	74.7%	P = 0.014	2.01 (1.12-3.62)	2	2.26 (0.81-6.29)	4	2.64 (1.31-5.34)	3	3.02 (1.11-8.26)	3	4.58 (1.12-18.78)
			(p = 0.001)										
**Post-trauma characteristics**													
Social support (yes)	1	0.21 (0.15-0.28)	-	-	-	1	0.21 (0.15-0.28)	1	0.21 (0.15-0.28)	1	0.21 (0.15-0.28)	1	0.21 (0.15-0.28)
Loss of property (yes)	5	0.97 (0.83-1.15)	14.0%	P = 0.037	-	3	0.92 (0.79-1.06)	5	0.97 (0.83-1.15)	2	1.25 (0.54-2.89)	1	1.02 (0.71-1.47)
			(p = 0.325)										
House damage (yes)	5	1.05 (0.84-1.32)	0%	P = 0.141	-	3	1.00 (0.76-1.34)	5	1.05 (0.84-1.32)	2	1.41 (0.87-2.27)	3	1.24 (0.90-1.69)
			(p = 0.500)										

The initial analysis of the trauma characteristics of survivors (i.e. before excluding low-quality and unadjusted studies) revealed that all five factors were associated with risk of depression; the pooled ORs were 1.73 (95% CI, 1.17–2.56) for being trapped, 2.39 (95% CI, 1.50–3.82) for experiencing fear, 2.60 (95% CI, 1.49–5.53) for experiencing injury, 1.68 (95% CI, 1.33–2.10) for witnessing injury/death, and 2.85 (95% CI, 1.59–5.11) for bereavement. However, after excluding low-quality and unadjusted studies, only two factors (bereavement and witnessing an injury or death) were still significantly associated with risk of depression.

Finally, with regard to the post-trauma characteristics of survivors, only one study found that social support was a protective factor for onset of depression (OR 0.21, 95% CI, 0.15–0.28).

## Discussion

To the best of our knowledge, this is the first meta-analysis focusing on risk factors for depression in populations specifically affected by natural disasters. Our synthesis of the relevant published English-language articles provided strong evidence for risk factors of depression following natural disasters. This study analyzed 31 published observational studies (1 case–control, 1 cohort, and 29 cross-sectional studies, including a total of 41,107 people). A total of 16 risk factors of depression in the survivors of natural disasters were explored in our study and categorized into three types: basic characteristics, trauma characteristics, and post-trauma characteristics.

With regard to basic characteristic, a common risk factor for the development of depression in both children and adults was prior exposure to trauma. This finding is consistent with previous research suggesting that the accumulation of violent traumatic events throughout the life course could increase the risk of depression [17,48]. Further common risk factors for adults were poor education, holding religious beliefs, and being female. Educational level indirectly influences economic resources, social status, social networks, health behaviour, and so on [49]. In natural disasters, people with higher education levels might use better coping methods because they have greater social resources, thus reducing the incidence of depression. Another interesting finding was that adults with religious beliefs were more susceptible to depression. Some research does substantiate our finding – for instance, Buddhists appeared to experience poorer mental health in the aftermath of the Wenchuan earthquake compared to people without religious beliefs [50]. This result might partly be explained by the belief that natural disasters are a punishment from God; this might lead to an increase in negative feelings such as guilt and depression [51].

Women were more likely to be depressed following a natural disaster than men were. Previous studies have indicated that women are more sensitive to threats, less likely to use effective coping strategies, and tend to interpret disasters more negatively than men do [9]. In addition, women are thought to be more sensitive to stress hormones, so their ability to manage stressful situations may be relatively poorer than men’s ability [22]. We did not find any gender difference among children in this meta-analysis. This may partly be explained by the fact that gender-specific characteristics have not yet fully developed during childhood [23]. We also found that married people are comparatively less harmed by natural disasters than were people who were unmarried, divorced, or widowed. Information about formal relationship status may potentially identify individuals with limited support structures and associated risk, offering useful directions for mental health monitoring and outreach programs [3].

In terms of trauma characteristics, children and adults shared three risk factors: experiencing fear, injury, and bereavement. It is likely that fear per se does not increase risk of depression; this effect is perhaps mediated instead by subjective experience of a natural disaster and personality type [10]. For example, individuals with high neuroticism tend to be more reactive and sensitive to adverse events, possibly increasing their risk of developing depression. The link between being injured and depression is possibly related to the severity of the injuries; injuries after a natural disaster are often so severe that they result in amputation and disability [21]. The onset of disability is likely to reduce quality of life in some people, and this loss of quality of life might lead to depression. Other studies have also demonstrated this link between physical injury and depression. Injuries that influence emotional and behavioural well-being may particularly contribute to the onset of depression [52,53]. Finally, bereavement after natural disasters was a risk factor for depression in both adult and child samples. Previous studies have indicated that bereavement in childhood is a potential risk factor for subsequent psychopathology [54]; furthermore, the extent of loss of family members is highly correlated with the incidence of depression, especially for children [55].

Children were more likely to develop depression if they were trapped or witnessed injury or death during the disaster. Being trapped is likely to generate a traumatic memory directly associated with the negative event, which is considered an important precursor to depression. It is unsurprising that children who witnessed someone being njured or killed during a disaster may have experienced intense fear during this time; as described above, fear can itself be a predictor of depression.

With regard to post-trauma characteristics, lower levels of social support were associated with a higher risk of depression in children. Lack of care and support from others may foster feelings of inferiority and insecurity among children, which act as catalysts for the development of depression [56]. However, this relationship was not found among adults. Modes of thinking are more complex in adults and so social support may not be as effective in relieving their negative moods after natural disasters [11]. However, only one study included in the meta-analysis investigated the relationship between social support and depression in children, so it is difficult to make generalisations without further research on this issue.

Adults who were unemployed were more likely to exhibit depressive symptoms after a natural disaster. This suggests that a loss of resources results in people being unable to care for their families to the extent that they could before the disaster [17,57]. Loss of property and house damage were also potential predictive factors for adult depression, indicating the need to examine the effects of socioeconomic conditions after the disaster on depression.

There are several potential limitations to our meta-analysis. First, we included only observational studies, which can be prone to biases in sample selection, recall, and information evaluation, as well as confounding bias. Second, the majority of the studies included in the meta-analysis (21 studies; 67.7%), were based on risk factors for depression specifically after an earthquake. Thus, it is premature to apply our results to survivors of all types of natural disasters. Finally, many of the variables included in the analysis were only examined in a small proportion of studies, which restricts the generalizability of the findings. Nevertheless, our study helps to highlight areas that would benefit from further investigation.

## Conclusions

In conclusion, our study demonstrated several risk factors for depression in children and adults following natural disasters. Despite the methodological limitations of the studies that we included in the meta-analysis, these findings are valuable for understanding how to reduce symptoms of depression following a natural disaster. Such research may provide clear intervention directions and result in development of psychosocial support programs for at-risk groups, or assist in the development of prevention programs for depression. General practitioners should be aware of depressive symptoms, and careful consideration should be given to routine screens for depression during the reconstruction process following natural disasters. Further research will also be required to determine suitable interventions for improving the mental health conditions of survivors in areas affected by natural disasters. Above all, post-disaster mental health recovery programs that include early identification, on-going monitoring, preventive and intervention programs, and sustained psychosocial support are needed for the high-risk population of natural disaster survivors.

## Abbreviations

NOS: Newcastle-ottawa scale; AHRQ: Agency for healthcare research and quality; OR: Odds ratio; RR: Relative risk.

## Competing interests

The authors declare that they have no competing interests.

## Authors’ contributions

BT, XL and LZ discussed and developed the question for this review. BT and XL carried out the searches. BT and XL assessed the eligibility of the studies for inclusion, extracted data and carried out all analysis. All authors were involved in interpreted and discussed results. BT wrote the first draft of this paper and it was reviewed by XL and LZ All authors agreed on the final draft of this study. LZ is the guarantor.

## Authors’ information

Bihan Tang and Xu Liu are co-first authors of this article.

## Pre-publication history

The pre-publication history for this paper can be accessed here:

http://www.biomedcentral.com/1471-2458/14/623/prepub

## Supplementary Material

Additional file 1 Moose statement - reporting checklist for authors, editors, and reviewers of meta-analyses of observational studies. Click here for file

Additional file 2: Table S1 Electronic databases and search query. Click here for file

Additional file 3: Figure S1 Search results and excluded/Included studies. Click here for file

Additional file 4: Table S2 Excluded studies and reasons for exclusion. Click here for file
