# Supplementary material for: A meta-analysis of risk factors for depression in adults and children after natural disasters
Source: BMC Public Health. 2014 Jun 19;14:623. doi: 10.1186/1471-2458-14-623 (PMC4077641; doi:10.1186/1471-2458-14-623)
Supplement: Additional file 1 — Moose statement - reporting checklist for authors, editors, and reviewers of meta-analyses of observational studies. [file 1471-2458-14-623-S1.docx]

**A meta-analysis of risk factors for depression in adults and children after natural disasters**

Bihan Tang^†^1, M.D.， Xu Liu^†^1, Ph.D.， Yuan Liu1, Ph.D.， Chen Xue1, Ph.D.， Lulu Zhang*1, Ph.D. Director and professor

**Author Affiliations:**

1Institute of Military Health Management, Second Military Medical University, Shanghai, China

^†^These authors contributed equally and are co-first authors of this article.

***Corresponding author:** Lulu Zhang, Director, Institute of Military Health Management, Second Military Medical University, 800 Xiangyin Rd, Shanghai, China, 200433

Tel: +86 021 81871421, Fax: +86 021 81871436, E-mail: zllsmmu@126.com

**Emails of all of the authors:**

Bihan Tang :mangotangbihan@126.com;

Xu Liu :aqualau@126.com;

Yuan Liu :yawnlau@126.com;

Chen Xue :xuechen8990@163.com;

Lulu Zhang :zllsmmu@126.com.

**Additional file 1**

**MOOSE Statement - Reporting Checklist for Authors, Editors, and Reviewers of Meta-analyses of Observational Studies**

| **Reporting Criteria** | **Reported (Yes/No)** | **Reported on Page** |
| --- | --- | --- |
| **Reporting of background should include** | | |
| Problem definition | Yes | 4 |
| Hypothesis statement | Yes | 4 |
| Description of study outcomes | Yes | 5 |
| Type of exposure or intervention used | Yes | 8 |
| Type of study designs used | Yes | 5 & Table 1 |
| Study population | Yes | 6 & Table 1 |
| **Reporting of search strategy should include** | | |
| Qualifications of searchers (eg librarians and investigators) | Yes | 5 |
| Search strategy, including time period used in the synthesis and key words | Yes | 5 |
| Effort to include all available studies, including contact with authors | YES | 6 |
| Databases and registries searched | YES | 5 |
| Search software used, name and version, including special features used (eg explosion) | NO |  |
| Use of hand searching (eg reference lists of obtained articles) | YES | 6 |
| List of citations located and those excluded, including justification | YES | 7 |
| Method of addressing articles published in languages other than English | NA |  |
| Method of handling abstracts and unpublished studies | NO |  |
| Description of any contact with authors | YES | 6 |
| **Reporting of methods should include** | | |
| Description of relevance or appropriateness of studies assembled for assessing the hypothesis to be tested | YES | 5 |
| Rationale for the selection and coding of data (eg sound clinical principles or convenience) | YES | 6 |
| Documentation of how data were classified and coded (eg multiple raters, blinding and interrater reliability) | YES | 6 |
| Assessment of confounding (eg comparability of cases and controls in studies where appropriate) | YES | 8 |
| Assessment of study quality, including blinding of quality assessors, stratification or regression on possible predictors of study results | YES | 7 |
| Assessment of heterogeneity | YES | 8 |
| Description of statistical methods (eg complete description of fixed or random effects models, justification of whether the chosen models account for predictors of study results, dose-response models, or cumulative meta-analysis) in sufficient detail to be replicated | YES | 8 |
| Provision of appropriate tables and graphics | YES | Table1-2 & Figure 1-2 |
| **Reporting of results should include** | | |
| Graphic summarizing individual study estimates and overall estimate | YES | Figure 1-2 |
| Table giving descriptive information for each study included | YES | Table1 |
| Results of sensitivity testing (eg subgroup analysis) | YES | 9-11 & Table 2 |
| Indication of statistical uncertainty of findings | YES | 9-11 |
| **Reporting of discussion should include** | | |
| Quantitative assessment of bias (eg publication bias) | YES | 9-11 |
| Justification for exclusion (eg exclusion of non-English language citations) | YES | 10 |
| Assessment of quality of included studies | YES | 11 |
| Strengths and weaknesses | YES | 15 |
| **Reporting of conclusions should include** | | |
| Consideration of alternative explanations for observed results | YES | 15 |
| Generalization of the conclusions (eg appropriate for the data presented and within the domain of the literature review) | YES | 15 |
| Guidelines for future research | YES | 15 |
| Disclosure of funding source | YES | 15 |

NA: Not Applicable
