# Supplementary material for: A meta-analysis of risk factors for depression in adults and children after natural disasters
Source: BMC Public Health. 2014 Jun 19;14:623. doi: 10.1186/1471-2458-14-623 (PMC4077641; doi:10.1186/1471-2458-14-623)
Supplement: Additional file 2: Table S1 — Electronic databases and search query. [file 1471-2458-14-623-S2.docx]

Additional file 2: Table S1 Electronic databases and search query

| Electronic databases | Search query | Results |
| --- | --- | --- |
| PubMed | ("mental disorders"[MeSH Terms] OR "mental disorders"[Title/Abstract] OR "mental disorder"[Title/Abstract] OR depress[Title/Abstract] OR depressed[Title/Abstract] OR depression[Title/Abstract] OR depressions[Title/Abstract] OR depressive[Title/Abstract] OR "Depression"[Mesh] OR "Depressive Disorder"[Mesh]) AND (earthquake[Title/Abstract] OR earthquakes[Title/Abstract] OR "earthquakes"[Mesh] OR tsunami[Title/Abstract] OR tsunamis[Title/Abstract] OR "tsunamis"[Mesh] OR flood[Title/Abstract] OR floods[Title/Abstract] OR "Floods"[Mesh] OR typhoon[Title/Abstract] OR typhoons[Title/Abstract] OR "Cyclonic Storms"[Mesh] OR hurricane[Title/Abstract] OR hurricanes[Title/Abstract] OR "volcanic eruptions"[Title/Abstract] OR "volcanic eruption"[Title/Abstract] OR "Volcanic Eruptions"[Mesh] OR mudslides[Title/Abstract] OR mudslide[Title/Abstract] OR "Landslides"[Mesh] OR fire[Title/Abstract] OR fires[Title/Abstract] OR "fires"[Mesh] OR Tornado[Title/Abstract] OR Tornadoes[Title/Abstract] OR "Tornadoes"[Mesh] OR Drought[Title/Abstract] OR Droughts[Title/Abstract] OR "Droughts"[Mesh] OR "natural disaster"[All Fields] OR "natural disasters"[All Fields]) | 2736 |
| Embase | ('earthquake'/exp OR 'tsunami'/exp OR 'flooding'/exp OR 'hurricane'/exp OR 'landslide'/exp OR ' drought '/exp OR 'natural disaster'/exp) AND ('depression'/exp OR 'mental disease'/exp) | 1612 |
| Web of Science | (TITLE: (earthquake$) OR TITLE: (natural disaster$)OR TITLE: (tsunami$) OR TITLE: (hurricane$) OR TITLE: (typhoon$) OR TITLE: (flood$) OR TITLE: (volcanic eruption$) OR TITLE: (drought$) OR TITLE: (tornado) OR TITLE: (fire$)) AND (TOPIC:(depression) OR TOPIC: (depressive) OR TOPIC: (mental disorder$)) | 1604 |
| PsycINFO | (Abstract: earthquake OR Abstract: (tsunami) OR Abstract: (hurricane) OR Abstract: (typhoon) OR Abstract: (flood) OR Abstract: (volcanic eruption) OR Abstract: (fire)) AND Abstract:(depression) | 15 |
