# Supplementary material for: A meta-analysis of risk factors for depression in adults and children after natural disasters
Source: BMC Public Health. 2014 Jun 19;14:623. doi: 10.1186/1471-2458-14-623 (PMC4077641; doi:10.1186/1471-2458-14-623)
Supplement: Additional file 3: Figure S1 — Search results and excluded/Included studies. [file 1471-2458-14-623-S3.doc]

2736 records identified in Pubmed

1612 records identified in Embase

1604 records identified in Web of Science

15 records identified in PsychInfo

1684 records excluded for duplication

4104 records excluded as they did not report information on risk factors for depression after natural disasters

148 articles excluded

92 records not having information on risk factors for depression after natural disasters

13 records based on people susceptible to depression

8 records not published in English

35 records had not effect size

179 full-text articles retrieved for further assessment

31 studies included in meta-analysis

20 studies were included in meta-analysis of risk factors for depression after natural disasters in adults

11 studies were included in meta-analysis of risk factors for depression after natural disasters in juveniles

Additional file 3: Figure S1. Search results and excluded/Included studies.
