# Supplementary material for: A meta-analysis of risk factors for depression in adults and children after natural disasters
Source: BMC Public Health. 2014 Jun 19;14:623. doi: 10.1186/1471-2458-14-623 (PMC4077641; doi:10.1186/1471-2458-14-623)
Supplement: Additional file 4: Table S2 — Excluded studies and reasons for exclusion. [file 1471-2458-14-623-S4.docx]

Additional file 4: Table S2 Excluded studies and reasons for exclusion

| Excluded studies | Reasons for exclusion |
| --- | --- |
| Ying[^1^](#_ENREF_1) | Did not reported OR/RR |
| Tsutsui^[2](#_ENREF_2)^ | Did not report information on risk factors for depression |
| Qu^[3](#_ENREF_3)^ | Was continuous depression score(no OR/RR) |
| Musa[^4^](#_ENREF_4) | Did not reported OR/RR |
| Kukihara^[5](#_ENREF_5)^ | Did not report information on risk factors for depression |
| Iwadare^[6](#_ENREF_6)^ | Did not report information on risk factors for depression |
| Cenat^[7](#_ENREF_7)^ | Was continuous depression score(no OR/RR) |
| Lai[^8^](#_ENREF_8) | Was continuous depression score(no OR/RR) |
| Guimaro^[9](#_ENREF_9)^ | Did not report information on risk factors for depression |
| Ying[^10^](#_ENREF_10) | Was continuous depression score(no OR/RR) |
| Xu^[11](#_ENREF_11)^ | Was continuous depression score(no OR/RR) |
| van der Velden^[12](#_ENREF_12)^ | Did not include risk factors for depression which we studied |
| Sullivan[^13^](#_ENREF_13) | Focused on people previously suffering from mental illness |
| Musa[^14^](#_ENREF_14) | Did not reported OR/RR |
| Liu[^15^](#_ENREF_15) | Focused on bereaved parents |
| Kobetz^[16](#_ENREF_16)^ | Did not report information on risk factors for depression |
| Joseph[^17^](#_ENREF_17) | Did not include risk factors for depression which we studied |
| Hirth^[18](#_ENREF_18)^ | Was continuous depression score(no OR/RR) |
| Feder^[19](#_ENREF_19)^ | Did not report corresponding 95% confidence intervals (CIs) |
| Berenz^[20](#_ENREF_20)^ | Did not include risk factors for depression which we studied |
| Bei^[21](#_ENREF_21)^ | Did not report information on risk factors for depression |
| Zhang[^22^](#_ENREF_22) | Was continuous depression score(no OR/RR) |
| Xu^[23](#_ENREF_23)^ | Did not report information on risk factors for depression |
| Wagenaar^[24](#_ENREF_24)^ | Was continuous depression score(no OR/RR) |
| Stratta^[25](#_ENREF_25)^ | Did not report information on risk factors for depression |
| Solangi^[26](#_ENREF_26)^ | Did not report information on risk factors for depression |
| Shultz[^27^](#_ENREF_27) | Did not report information on risk factors for depression |
| Sezgin^[28](#_ENREF_28)^ | Was continuous depression score(no OR/RR) |
| Ruggiero[^29^](#_ENREF_29) | Did not reported OR/RR |
| Raguenaud^[30](#_ENREF_30)^ | Did not report information on risk factors for depression |
| Qu^[31](#_ENREF_31)^ | Was ordinal depression score |
| Qu^[32](#_ENREF_32)^ | Focused on pregnant women |
| Qu^[33](#_ENREF_33)^ | Focused on new mothers |
| Pietrzak^[34](#_ENREF_34)^ | Did not report corresponding 95% CIs |
| Kawada[^35^](#_ENREF_35) | Did not report information on risk factors for depression |
| Gros^[36](#_ENREF_36)^ | Was continuous depression score(no OR/RR) |
| Gigantesco^[37](#_ENREF_37)^ | Did not report information on risk factors for depression |
| Aziz[^38^](#_ENREF_38) | Did not report information on risk factors for depression |
| Papadatou^[39](#_ENREF_39)^ | Did not reported OR/RR |
| Papanikolaou^[40](#_ENREF_40)^ | Was continuous depression score(no OR/RR) |
| Zhu[^41^](#_ENREF_41) | Did not publish in English |
| Wind[^42^](#_ENREF_42) | Did not report information on risk factors for depression |
| Wickrama^[43](#_ENREF_43)^ | Did not report information on risk factors for depression |
| Suzuki[^44^](#_ENREF_44) | Did not report information on risk factors for depression |
| Rosendal^[45](#_ENREF_45)^ | Was continuous depression score(no OR/RR) |
| Pollice^[46](#_ENREF_46)^ | Did not report information on risk factors for depression |
| Marres^[47](#_ENREF_47)^ | Did not report information on risk factors for depression |
| Kilic^[48](#_ENREF_48)^ | Did not report information on risk factors for depression |
| Hussain^[49](#_ENREF_49)^ | Did not report information on risk factors for depression |
| Harville^[50](#_ENREF_50)^ | Was continuous depression score(no OR/RR) |
| Ehring^[51](#_ENREF_51)^ | Was continuous depression score(no OR/RR) |
| Xiong^[52](#_ENREF_52)^ | Focused on pregnant women |
| Murphy[^53^](#_ENREF_53) | Did not report information on risk factors for depression |
| Mason[^54^](#_ENREF_54) | Did not include risk factors for depression which we studied |
| Kronenberg^[55](#_ENREF_55)^ | Did not report information on risk factors for depression |
| Khan[^56^](#_ENREF_56) | Did not report information on risk factors for depression |
| Huang[^57^](#_ENREF_57) | Did not publish in English |
| Harville^[58](#_ENREF_58)^ | Did not report information on risk factors for depression |
| Ehrlich[^59^](#_ENREF_59) | Focused on pregnant women |
| Davis[^60^](#_ENREF_60) | Did not report information on risk factors for depression |
| Bailey[^61^](#_ENREF_61) | Did not report information on risk factors for depression |
| Wei[^62^](#_ENREF_62) | Did not publish in English |
| Wadsworth[^63^](#_ENREF_63) | Did not report information on risk factors for depression |
| Udomratn^[64](#_ENREF_64)^ | Did not report information on risk factors for depression |
| Thavichachart^[65](#_ENREF_65)^ | Did not report information on risk factors for depression |
| Scher^[66](#_ENREF_66)^ | Did not report information on risk factors for depression |
| Tees[^67^](#_ENREF_67) | Focused on pregnant women |
| Suzuki[^68^](#_ENREF_68) | Did not publish in English |
| Nandi[^69^](#_ENREF_69) | Did not report information on risk factors for depression |
| Kristensen^[70](#_ENREF_70)^ | Did not report information on risk factors for depression |
| Kraemer[^71^](#_ENREF_71) | Did not report information on risk factors for depression |
| Harville^[72](#_ENREF_72)^ | Focused on pregnant women |
| Harville^[73](#_ENREF_73)^ | Did not report information on risk factors for depression |
| Gul^[74](#_ENREF_74)^ | Did not report information on risk factors for depression |
| Goenjian^[75](#_ENREF_75)^ | Did not reported OR/RR |
| Fernando[^76^](#_ENREF_76) | Did not report information on risk factors for depression |
| Xiong^[77](#_ENREF_77)^ | Did not report information on risk factors for depression |
| Wickrama^[78](#_ENREF_78)^ | Focused on new mothers |
| Wang[^79^](#_ENREF_79) | Did not publish in English |
| Vigil[^80^](#_ENREF_80) | Did not report information on risk factors for depression |
| Ularntinon^[81](#_ENREF_81)^ | Did not report information on risk factors for depression |
| Scaramella^[82](#_ENREF_82)^ | Did not report information on risk factors for depression |
| Kuwabara^[83](#_ENREF_83)^ | Did not report information on risk factors for depression |
| Heo^[84](#_ENREF_84)^ | Did not report information on risk factors for depression |
| David[^85^](#_ENREF_85) | Did not report information on risk factors for depression |
| Piyavhatkul^[86](#_ENREF_86)^ | Did not report information on risk factors for depression |
| Piyasil^[87](#_ENREF_87)^ | Did not report information on risk factors for depression |
| Pina^[88](#_ENREF_88)^ | Did not include risk factors for depression which we studied |
| Kuwabara^[89](#_ENREF_89)^ | Did not report information on risk factors for depression |
| Hunt[^90^](#_ENREF_90) | Did not report information on risk factors for depression |
| Goenjian^[91](#_ENREF_91)^ | Did not report information on risk factors for depression |
| Duan^[92](#_ENREF_92)^ | Did not publish in English |
| Xiong^[93](#_ENREF_93)^ | Focused on pregnant women |
| Wickrama^[94](#_ENREF_94)^ | Did not report information on risk factors for depression |
| Souza[^95^](#_ENREF_95) | Did not report information on risk factors for depression |
| Silove^[96](#_ENREF_96)^ | Did not report information on risk factors for depression |
| Prueksaritanond^[97](#_ENREF_97)^ | Did not reported OR/RR |
| James[^98^](#_ENREF_98) | Did not report information on risk factors for depression |
| Harville^[99](#_ENREF_99)^ | Focused on pregnant women |
| Beaudoin^[100](#_ENREF_100)^ | Did not include risk factors for depression which we studied |
| Seplaki^[101](#_ENREF_101)^ | Was continuous depression score(no OR/RR) |
| Kar^[102](#_ENREF_102)^ | Did not report information on risk factors for depression |
| Yazgan^[103](#_ENREF_103)^ | Did not report information on risk factors for depression |
| Seplaki^[104](#_ENREF_104)^ | Did not report information on risk factors for depression |
| Sattler[^105^](#_ENREF_105) | Did not report information on risk factors for depression |
| Kisac^[106](#_ENREF_106)^ | Did not report information on risk factors for depression |
| Kilic^[107](#_ENREF_107)^ | Did not reported OR/RR |
| Giannopoulou^[108](#_ENREF_108)^ | Did not reported OR/RR |
| Aksaray^[109](#_ENREF_109)^ | Did not reported OR/RR |
| Aker[^110^](#_ENREF_110) | Was a review |
| Acierno^[111](#_ENREF_111)^ | Did not reported OR/RR |
| Roussos[^112^](#_ENREF_112) | Did not reported OR/RR |
| Montazeri^[113](#_ENREF_113)^ | Did not report information on risk factors for depression |
| Chang[^114^](#_ENREF_114) | Did not report information on risk factors for depression |
| Livanou^[115](#_ENREF_115)^ | Did not report information on risk factors for depression |
| Kohn[^116^](#_ENREF_116) | Did not report information on risk factors for depression |
| Kohn[^117^](#_ENREF_117) | Did not report information on risk factors for depression |
| Altindag^[118](#_ENREF_118)^ | Did not report information on risk factors for depression |
| Desai[^119^](#_ENREF_119) | Did not report information on risk factors for depression |
| Watanabe[^120^](#_ENREF_120) | Did not report information on risk factors for depression |
| Norris[^121^](#_ENREF_121) | Did not report information on risk factors for depression |
| Chou[^122^](#_ENREF_122) | Did not report information on risk factors for depression |
| Kuo^[123](#_ENREF_123)^ | Did not report information on risk factors for depression |
| Kolaitis^[124](#_ENREF_124)^ | Did not report information on risk factors for depression |
| Kilic^[125](#_ENREF_125)^ | Did not reported OR/RR |
| Livanou^[126](#_ENREF_126)^ | Did not report information on risk factors for depression |
| Liao[^127^](#_ENREF_127) | Focused on rescue workers |
| Basoglu^[128](#_ENREF_128)^ | Did not report information on risk factors for depression |
| Najarian^[129](#_ENREF_129)^ | Did not report information on risk factors for depression |
| Maruyama[^130^](#_ENREF_130) | Did not report information on risk factors for depression |
| Goenjian^[131](#_ENREF_131)^ | Did not reported OR/RR |
| Shioyama^[132](#_ENREF_132)^ | Did not report information on risk factors for depression |
| Goenjian^[133](#_ENREF_133)^ | Did not report information on risk factors for depression |
| McDermott[^134^](#_ENREF_134) | Was continuous depression score(no OR/RR) |
| Watson[^135^](#_ENREF_135) | Focused on prenatal teratogens |
| Sharan^[136](#_ENREF_136)^ | Did not report information on risk factors for depression |
| Najarian^[137](#_ENREF_137)^ | Did not report information on risk factors for depression |
| Kato[^138^](#_ENREF_138) | Did not report information on risk factors for depression |
| David[^139^](#_ENREF_139) | Did not reported OR/RR |
| Karanci^[140](#_ENREF_140)^ | Did not report information on risk factors for depression |
| Goenjian^[141](#_ENREF_141)^ | Did not report information on risk factors for depression |
| Hardin[^142^](#_ENREF_142) | Did not reported OR/RR |
| Thompson[^143^](#_ENREF_143) | Did not report information on risk factors for depression |
| Jeney-Gammon[^144^](#_ENREF_144) | Did not report information on risk factors for depression |
| Lima[^145^](#_ENREF_145) | Did not publish in English |
| Shore[^146^](#_ENREF_146) | Did not report information on risk factors for depression |
| Murphy[^147^](#_ENREF_147) | Did not report information on risk factors for depression |
| Grecu[^148^](#_ENREF_148) | Did not publish in English |

.

**Reference**

1. Ying L, Wu X, Lin C, Jiang L. Traumatic severity and trait resilience as predictors of posttraumatic stress disorder and depressive symptoms among adolescent survivors of the Wenchuan earthquake. *PLoS ONE* 2014;9(2).

2. Tsutsui T, Hasegawa Y, Hiraga M, Ishiki M, Asukai N. Distinctiveness of prolonged grief disorder symptoms among survivors of the Great East Japan Earthquake and Tsunami. *Psychiatry Research* 2014.

3. Qu Z, Wang C-W, Zhang X, Ho AHY, Wang X, Chan CLW. Prevalence and determinants of depression among survivors 8 months after the wenchuan earthquake. *The Journal of nervous and mental disease* 2014;202(4):275-9.

4. Musa R, Draman S, Jeffrey S, Jeffrey I, Abdullah N, Halim NAM, et al. Post tsunami psychological impact among survivors in Aceh and West Sumatra, Indonesia. *Comprehensive Psychiatry* 2014;55 Suppl 1:S13-6.

5. Kukihara H, Yamawaki N, Uchiyama K, Arai S, Horikawa E. Trauma, depression, and resilience of earthquake/tsunami/nuclear disaster survivors of hirono, fukushima, japan. *Psychiatry and Clinical Neurosciences* 2014.

6. Iwadare Y, Usami M, Suzuki Y, Ushijima H, Tanaka T, Watanabe K, et al. Posttraumatic Symptoms in Elementary and Junior High School Children after the 2011 Japan Earthquake and Tsunami: Symptom Severity and Recovery Vary by Age and Sex. *Journal of Pediatrics* 2014;164(4):917-U307.

7. Cenat JM, Derivois D. Assessment of prevalence and determinants of posttraumatic stress disorder and depression symptoms in adults survivors of earthquake in Haiti after 30 months. *Journal of Affective Disorders* 2014;159:111-17.

8. Lai BS, La Greca AM, Auslander BA, Short MB. Children's symptoms of posttraumatic stress and depression after a natural disaster: Comorbidity and risk factors. *Journal of Affective Disorders* 2013;146(1):71-78.

9. Guimaro MS, Steinman M, Kernkraut AM, Santos OFPd, Lacerda SS. Psychological distress in survivors of the 2010 Haiti earthquake. *Einstein (Sao Paulo, Brazil)* 2013;11(1):11-4.

10. Ying Lh, Wu Xc, Lin Cd, Chen C. Prevalence and predictors of posttraumatic stress disorder and depressive symptoms among child survivors 1 year following the Wenchuan earthquake in China. *European Child and Adolescent Psychiatry* 2013:1-9.

11. Xu J, Mo L, Wu Z. A Cross-Sectional Study on Risk Factors of Depression Severity Among Survivors of the 2008 Sichuan Earthquake. *Community Ment Health J* 2013.

12. van der Velden PG, Wong A, Boshuizen HC, Grievink L. Persistent mental health disturbances during the 10years after a disaster: Four-wave longitudinal comparative study. *Psychiatry and Clinical Neurosciences* 2013;67(2):110-18.

13. Sullivan G, Vasterling JJ, Han XT, Tharp AT, Davis T, Deitch EA, et al. Preexisting Mental Illness and Risk for Developing a New Disorder After Hurricane Katrina. *Journal of Nervous and Mental Disease* 2013;201(2):161-66.

14. Musa R, Draman S, Jeffrey S, Jeffrey I, Abdullah N, Halim NA, et al. Post tsunami psychological impact among survivors in Aceh and West Sumatra, Indonesia. *Compr Psychiatry* 2013.

15. Liu W, Fan F, Liu J. Depressive symptoms in bereaved parents in the 2008 wenchuan, china earthquake: a cohort study. *J Trauma Stress* 2013;26(2):274-9.

16. Kobetz E, Menard J, Kish J, Bishop I, Hazan G, Nicolas G. Impacts of the 2010 Haitian earthquake in the diaspora: findings from Little Haiti, Miami, FL. *Journal of immigrant and minority health / Center for Minority Public Health* 2013;15(2):442-7.

17. Joseph NT, Matthews KA, Myers HF. Conceptualizing Health Consequences of Hurricane Katrina From the Perspective of Socioeconomic Status Decline. *Health psychology : official journal of the Division of Health Psychology, American Psychological Association* 2013.

18. Hirth JM, Leyser-Whalen O, Berenson AB. Effects of a major u.s. Hurricane on mental health disorder symptoms among adolescent and young adult females. *The Journal of adolescent health : official publication of the Society for Adolescent Medicine* 2013;52(6):765-72.

19. Feder A, Ahmad S, Lee EJ, Morgan JE, Singh R, Smith BW, et al. Coping and PTSD symptoms in Pakistani earthquake survivors: Purpose in life, religious coping and social support. *Journal of Affective Disorders* 2013;147(1-3):156-63.

20. Berenz EC, Trapp SK, Acierno R, Richardson L, Kilpatrick DG, Trinh Luong T, et al. PRETYPHOON PANIC ATTACK HISTORY MODERATES THE RELATIONSHIP BETWEEN DEGREE OF TYPHOON EXPOSURE AND POSTTYPHOON PTSD AND DEPRESSION IN A VIETNAMESE SAMPLE. *Depression and Anxiety* 2013;30(5):461-68.

21. Bei B, Bryant C, Gilson K-M, Koh J, Gibson P, Komiti A, et al. A prospective study of the impact of floods on the mental and physical health of older adults. *Aging & mental health* 2013;17(8):992-1002.

22. Zhang Z, Ran MS, Li YH, Ou GJ, Gong RR, Li RH, et al. Prevalence of post-traumatic stress disorder among adolescents after the Wenchuan earthquake in China. *Psychological medicine* 2012;42(8):1687-93.

23. Xu J, He Y. Psychological health and coping strategy among survivors in the year following the 2008 Wenchuan earthquake. *Psychiatry Clin Neurosci* 2012;66(3):210-9.

24. Wagenaar BH, Hagaman AK, Kaiser BN, McLean KE, Kohrt BA. Depression, suicidal ideation, and associated factors: a cross-sectional study in rural Haiti. *BMC Psychiatry* 2012;12.

25. Stratta P, de Cataldo S, Bonanni R, Valenti M, Masedu F, Rossi A. Mental health in L'Aquila after the earthquake. *Annali dell'Istituto superiore di sanita* 2012;48(2):132-7.

26. Solangi M, Zafar S, Moeezudin M, Zafar I, Rehman RU. Psychiatric morbidity among internally displaced persons (idps) of sindh. *European Psychiatry* 2012;27.

27. Shultz JM, Besser A, Kelly F, Allen A, Schmitz S, Hausmann V, et al. Psychological consequences of indirect exposure to disaster due to the haiti earthquake. *Prehospital and Disaster Medicine* 2012;27(4):359-68.

28. Sezgin U, Punamaki RL. Earthquake trauma and causal explanation associating with PTSD and other psychiatric disorders among South East Anatolian women. *Journal of Affective Disorders* 2012;141(2-3):432-40.

29. Ruggiero KJ, Gros K, McCauley JL, Resnick HS, Morgan M, Kilpatrick DG, et al. Mental health outcomes among adults in Galveston and Chambers counties after Hurricane Ike. *Disaster Med Public Health Prep* 2012;6(1):26-32.

30. Raguenaud ME, Germonneau P, Leseigneur J, Chavagnat JJ, Motreff Y, Vivier-Darrigol M, et al. Epidemiological surveillance linked to an outreach psychological support program after the Xynthia storm in Charente-Maritime, France, 2010. *Prehosp Disaster Med* 2012;27(5):483-8.

31. Qu ZY, Wang XH, Tian DH, Zhao Y, Zhang Q, He H, et al. Posttraumatic stress disorder and depression among new mothers at 8 months later of the 2008 Sichuan earthquake in China. *Archives of Womens Mental Health* 2012;15(1):49-55.

32. Qu ZY, Tian DH, Zhang Q, Wang XH, He H, Zhang XL, et al. The impact of the catastrophic earthquake in China's Sichuan province on the mental health of pregnant women. *Journal of Affective Disorders* 2012;136(1-2):117-23.

33. Qu Z, Wang X, Tian D, Zhao Y, Zhang Q, He H, et al. Posttraumatic stress disorder and depression among new mothers at 8 months later of the 2008 Sichuan earthquake in China. *Archives of Women's Mental Health* 2012;15(1):49-55.

34. Pietrzak RH, Southwick SM, Tracy M, Galea S, Norris FH. Posttraumatic stress disorder, depression, and perceived needs for psychological care in older persons affected by Hurricane Ike. *Journal of Affective Disorders* 2012;138(1-2):96-103.

35. Kawada T. Risk assessment for earthquake survivors: evaluation of generation difference. *Disaster Med Public Health Prep* 2012;6(4):325.

36. Gros DF, Price M, Gros KS, Paul LA, McCauley JL, Ruggiero KJ. Relations between loss of services and psychiatric symptoms in urban and non-urban settings following a natural disaster. *Journal of Psychopathology and Behavioral Assessment* 2012;34(3):343-50.

37. Gigantesco A, Mirante N, Minardi V, Tarolla E, Cofini V, Carbonelli A, et al. [Depressive symptoms, a challenge for the community of L'Aquila after the earthquake of 2009]. *Epidemiologia e prevenzione* 2012;36(2):129.

38. Aziz S, Aslam N. Psychiatric Morbidity and Work and Social Adjustment Among Earthquake Survivors Extricated from under the Rubble. *Indian journal of psychological medicine* 2012;34(4):346-9.

39. Papadatou D, Giannopoulou I, Bitsakou P, Bellali T, Talias MA, Tselepi K. Adolescents' reactions after a wildfire disaster in Greece. *J Trauma Stress* 2012;25(1):57-63.

40. Papanikolaou V, Adamis D, Mellon RC, Prodromitis G. Psychological distress following wildfires disaster in a rural part of Greece: a case-control population-based study. *Int J Emerg Ment Health* 2011;13(1):11-26.

41. Zhu CZ, Situ MJ, Zhang Y, Fang H, Jing LS, Wang D, et al. [Influence factors of post-traumatic stress disorder (PTSD) and depression symptoms in children and adolescents after Wenchuan earthquake in China]. *Zhonghua yu fang yi xue za zhi [Chinese journal of preventive medicine]* 2011;45(6):531-36.

42. Wind TR, Fordham M, Komproe IH. Social capital and post-disaster mental health. *Glob Health Action* 2011;4.

43. Wickrama KA, Wickrama T. Perceived community participation in tsunami recovery efforts and the mental health of tsunami-affected mothers: findings from a study in rural Sri Lanka. *The International journal of social psychiatry* 2011;57(5):518-27.

44. Suzuki Y, Tsutsumi A, Fukasawa M, Honma H, Someya T, Kim Y. Prevalence of mental disorders and suicidal thoughts among community-dwelling elderly adults 3 years after the Niigata-Chuetsu earthquake. *Journal of Epidemiology* 2011;21(2):144-50.

45. Rosendal S, Salcioglu E, Andersen HS, Mortensen EL. Exposure characteristics and peri-trauma emotional reactions during the 2004 tsunami in Southeast Asia-what predicts posttraumatic stress and depressive symptoms? *Comprehensive Psychiatry* 2011;52(6):630-37.

46. Pollice R, Bianchini V, Marola V, Verni L, Di Mauro S, Ussorio D, et al. POST-TRAUMATIC AND PSYCHIATRIC SYMPTOMS AMONG YOUNG EARTHQUAKE SURVIVORS IN PRIMARY CARE CAMP HOSPITAL. *European Journal of Inflammation* 2011;9(1):39-44.

47. Marres GMH, Leenen LPH, de Vries J, Mulder PGH, Vermetten E. Disaster-related injury and predictors of health complaints after exposure to a natural disaster: an online survey. *BMJ Open* 2011;1(2).

48. Kilic C, Kilic EZ, Aydin IO. Effect of relocation and parental psychopathology on earthquake survivor-children's mental health. *The Journal of nervous and mental disease* 2011;199(5):335-41.

49. Hussain A, Weisaeth L, Heir T. Psychiatric disorders and functional impairment among disaster victims after exposure to a natural disaster: A population based study. *Journal of Affective Disorders* 2011;128(1-2):135-41.

50. Harville EW, Xiong X, Smith BW, Pridjian G, Elkind-Hirsch K, Buekens P. Combined effects of Hurricane Katrina and Hurricane Gustav on the mental health of mothers of small children. *Journal of Psychiatric and Mental Health Nursing* 2011;18(4):288-96.

51. Ehring T, Razik S, Emmelkamp PM. Prevalence and predictors of posttraumatic stress disorder, anxiety, depression, and burnout in Pakistani earthquake recovery workers. *Psychiatry Res* 2011;185(1-2):161-6.

52. Xiong X, Harville EW, Mattison DR, Elkind-Hirsch K, Pridjian G, Buekens P. Hurricane Katrina experience and the risk of post-traumatic stress disorder and depression among pregnant women. *American journal of disaster medicine* 2010;5(3):181-87.

53. Murphy SA. Women's and children's exposure to mass disaster and terrorist attacks. *Issues in mental health nursing* 2010;31(1):45-53.

54. Mason V, Andrews H, Upton D. The psychological impact of exposure to floods. *Psychology, health & medicine* 2010;15(1):61-73.

55. Kronenberg ME, Hansel TC, Brennan AM, Osofsky HJ, Osofsky JD, Lawrason B. Children of Katrina: Lessons learned about postdisaster symptoms and recovery patterns. *Child Development* 2010;81(4):1241-59.

56. Khan M, Masood M, Mukhtar M, Sana N, Chaudhry H. Gender differences in prevalence of anxiety disorders among earth quake survivors. *European Psychiatry* 2010;25.

57. Huang MJ, Guo LT, Li J, Sun XL, Zhang BZ, Yi QM, et al. [Epidemiological investigation on major depressive disorder in the most heavily damaged areas from Wenchuan earthquake in 2008]. *Zhonghua liu xing bing xue za zhi = Zhonghua liuxingbingxue zazhi* 2010;31(2):167-70.

58. Harville EW, Xiong X, Buekens P, Pridjian G, Elkind-Hirsch K. Resilience After Hurricane Katrina Among Pregnant and Postpartum Women. *Women's Health Issues* 2010;20(1):20-27.

59. Ehrlich M, Harville E, Xiong X, Buekens P, Pridjian G, Elkind-Hirsch K. Loss of resources and hurricane experience as predictors of postpartum depression among women in Southern Louisiana. *Journal of Women's Health* 2010;19(5):877-84.

60. Davis TE, Grills-Taquechel AE, Ollendick TH. The Psychological Impact From Hurricane Katrina: Effects of Displacement and Trauma Exposure on University Students. *Behavior Therapy* 2010;41(3):340-49.

61. Bailey RK, Bailey T, Akpudo H. On the ground in Haiti: a psychiatrist's evaluation of post earthquake Haiti. *J Health Care Poor Underserved* 2010;21(2):417-21.

62. Wei N, Zhou WH, Hu JB, Huang ML, Qi HL, Hu SH, et al. A survey of mental health among transferred injured survivors in Wenchuan earthquake in Sichuan. *Zhonghua yu fang yi xue za zhi [Chinese journal of preventive medicine]* 2009;43(5):376-79.

63. Wadsworth ME, de Carlo Santiago C, Einhorn L. Coping with displacement from Hurricane Katrina: Predictors of one-year post-traumatic stress and depression symptom trajectories. *Anxiety, Stress and Coping* 2009;22(4):413-32.

64. Udomratn P. Prevalence of tsunami-related PTSD and MDD in Thailand. *Asian J Psychiatr* 2009;2(4):124-7.

65. Thavichachart N, Tangwongchai S, Worakul P, Kanchanatawan B, Suppapitiporn S, Na Pattalung AS, et al. Posttraumatic stress disorder of the tsunami survivors in Thailand. *Journal of the Medical Association of Thailand* 2009;92(3):420-29.

66. Scher CD, Ellwanger J. Fire-related cognitions moderate the impact of risk factors on adjustment following wildfire disaster. *Journal of Anxiety Disorders* 2009;23(7):891-96.

67. Tees MT, Harville EW, Xiong X, Buekens P, Pridjian G, Elkind-Hirsch K. Hurricane Katrina-Related Maternal Stress, Maternal Mental Health, and Early Infant Temperament. *Maternal and Child Health Journal* 2009:1-8.

68. Suzuki Y, Honma H, Tsutsumi A, Kim Y. [Morbidity survey on community-residing aged individuals 3 years after Niidata-Chuetsu Earthquake]. *Seishin Shinkeigaku Zasshi* 2009;111(4):405-10.

69. Nandi A, Tracy M, Beard JR, Vlahov D, Galea S. Patterns and Predictors of Trajectories of Depression after an Urban Disaster. *Annals of Epidemiology* 2009;19(11):761-70.

70. Kristensen P, Weisaeth L, Heir T. Psychiatric disorders among disaster bereaved: An interview study of individuals directly or not directly exposed to the 2004 tsunami. *Depression and Anxiety* 2009;26(12):1127-33.

71. Kraemer B, Wittmann L, Jenewein J, Schnyder U. 2004 Tsunami: Long-term psychological consequences for Swiss tourists in the area at the time of the disaster. *Australian and New Zealand Journal of Psychiatry* 2009;43(5):420-25.

72. Harville EW, Xiong X, Pridjian G, Elkind-Hirsch K, Buekens P. Postpartum mental health after Hurricane Katrina: a cohort study. *BMC pregnancy and childbirth* 2009;9:21.

73. Harville EW, Xiong X, Buekens P. Hurricane katrina and perinatal health. *Birth* 2009;36(4):325-31.

74. Gul M, Faruqui R. Gender difference in parental psychiatric morbidity in the aftermath of 2005 South Asian earthquake. *European Psychiatry* 2009;24:S794.

75. Goenjian AK, Walling D, Steinberg AM, Roussos A, Goenjian HA, Pynoos RS. Depression and PTSD symptoms among bereaved adolescents 61/2 years after the 1988 spitak earthquake. *Journal of Affective Disorders* 2009;112(1-3):81-84.

76. Fernando G, Miller K, Garcia J, Prasad J, Suseema U, Laurin D, et al. Predictors of psychological functioning in Sri Lankan youth: Trauma exposure, perceived availability of resources, and perceived helpfulness of religious practices. *European Psychiatry* 2009;24:S1243.

77. Xiong X, Harville EW, Mattison DR, Elkind-Hirsch K, Pridjian G, Buekens P. Exposure to Hurricane Katrina, post-traumatic stress disorder and birth outcomes. *American Journal of the Medical Sciences* 2008;336(2):111-15.

78. Wickrama KA. Family context of mental health risk in Tsunami affected mothers: findings from a pilot study in Sri Lanka. *Social science & medicine (1982)* 2008;66(4):994-1007.

79. Wang J, Yang FD, Sun CY. The analysis of depression, anxiety and sleep disorder in earthquake-related injuries. *Zhonghua nei ke za zhi [Chinese journal of internal medicine]* 2008;47(9):721-22.

80. Vigil JM, Geary DC. A preliminary investigation of family coping styles and psychological well-being among adolescent survivors of Hurricane Katrina. *Journal of Family Psychology* 2008;22(1):176-80.

81. Ularntinon S, Piyasil V, Ketumarn P, Sitdhiraksa N, Pityaratstian N, Lerthattasilp T, et al. Assessment of psychopathological consequences in children at 3 years after tsunami disaster. *Journal of the Medical Association of Thailand = Chotmaihet thangphaet* 2008;91 Suppl 3:S69-75.

82. Scaramella LV, Sohr-Preston SL, Callahan KL, Mirabile SP. A test of the Family Stress Model on toddler-aged children's adjustment among Hurricane Katrina impacted and nonimpacted low-income families. *Journal of clinical child and adolescent psychology : the official journal for the Society of Clinical Child and Adolescent Psychology, American Psychological Association, Division 53* 2008;37(3):530-41.

83. Kuwabara H, Shioiri T, Toyabe S-I, Kawamura T, Koizumi M, Ito-Sawamura M, et al. Factors impacting on psychological distress and recovery after the 2004 Niigata-Chuetsu earthquake, Japan: Community-based study. *Psychiatry and Clinical Neurosciences* 2008;62(5):503-07.

84. Heo JH, Kim MH, Koh SB, Noh S, Park JH, Ahn JS, et al. A prospective study on changes in health status following flood disaster. *Psychiatry Investigation* 2008;5(3):186-92.

85. David M, Ceschi G, Billieux J, Van Der Linden M. Depressive symptoms after trauma: Is self-esteem a mediating factor? *Journal of Nervous and Mental Disease* 2008;196(10):735-42.

86. Piyavhatkul N, Pairojkul S, Suphakunpinyo C. Psychiatric disorders in tsunami-affected children in Ranong province, Thailand. *Medical Principles and Practice* 2008;17(4):290-95.

87. Piyasil V, Ketumarn P, Prubrukarn R, Pacharakaew S, Dumrongphol H, Rungsri S, et al. Psychiatric disorders in children at one year after the tsunami disaster in Thailand. *Journal of the Medical Association of Thailand = Chotmaihet thangphaet* 2008;91 Suppl 3:S15-20.

88. Pina AA, Villalta IK, Ortiz CD, Gottschall AC, Costa NM, Weems CF. Social support, discrimination, and coping as predictors of posttraumatic stress reactions in youth survivors of Hurricane Katrina. *Journal of Clinical Child and Adolescent Psychology* 2008;37(3):564-74.

89. Kuwabara H, Shioiri T, Toyabe SI, Kawamura T, Koizumi M, Ito-Sawamura M, et al. Factors impacting on psychological distress and recovery after the 2004 Niigata-Chuetsu earthquake, Japan: Community-based study. *Psychiatry and Clinical Neurosciences* 2008;62(5):503-07.

90. Hunt M, Al-Awadi H, Johnson M. Psychological sequelae of pet loss following Hurricane Katrina. *Anthrozoos* 2008;21(2):109-21.

91. Goenjian AK, Noble EP, Walling DP, Goenjian HA, Karayan IS, Ritchie T, et al. Heritabilities of symptoms of posttraumatic stress disorder, anxiety, and depression in earthquake exposed Armenian families. *Psychiatric Genetics* 2008;18(6):261-66.

92. Duan GF, Wang J, Zhou WM. Effect of mental nursing for operation patients with earthquake trauma on their rehabilitation. *Chinese Journal of Evidence-Based Medicine* 2008;8(10):820-22.

93. Xiong X, Harville EW, Buekens P, Mattison DR, Elkind-Hirsch K, Pridjian G. Post-traumatic stress disorder and depression in pregnant women affected by Hurricane Katrina. *American Journal of Epidemiology* 2007;165(11):S22-S22.

94. Wickrama KA, Kaspar V. Family context of mental health risk in Tsunami-exposed adolescents: findings from a pilot study in Sri Lanka. *Social science & medicine (1982)* 2007;64(3):713-23.

95. Souza R, Bernatsky S, Reyes R, de Jong K. Mental health status of vulnerable tsunami-affected communities: a survey in Aceh Province, Indonesia. *J Trauma Stress* 2007;20(3):263-9.

96. Silove DM. Increased prevalence of post-traumatic stress disorder, anxiety and depression in displaced tsunami survivors from southern Thailand. *Evidence-based mental health* 2007;10(1):31.

97. Prueksaritanond S, Kongsakol R. Biopsychosocial impacts on the elderly from a tsunami-affected community in southern Thailand. *Journal of the Medical Association of Thailand* 2007;90(8):1501-05.

98. James NT, Miller CW, Nugent K, Welch C, Cabanna M, Vincent S. The impact of Hurricane Katrina upon older adult nurses: an assessment of quality of life and psychological distress in the aftermath. *Journal of the Mississippi State Medical Association* 2007;48(10):299-307.

99. Harville EW, Xiong X, Buekens P, Elkind-Hirsch K, Pridjian G. Postpartum depression and post-traumatic stress disorder after Hurricane Katrina. *American Journal of Epidemiology* 2007;165(11):S71-S71.

100. Beaudoin CE. News, social capital and health in the context of Katrina. *Journal of Health Care for the Poor and Underserved* 2007;18(2):418-30.

101. Seplaki CL, Goldman N, Weinstein M, Lin Y-H. Before and after the 1999 Chi-Chi earthquake: traumatic events and depressive symptoms in an older population. *Social science & medicine (1982)* 2006;62(12):3121-32.

102. Kar N, Bastia BK. Post-traumatic stress disorder, depression and generalised anxiety disorder in adolescents after a natural disaster: a study of comorbidity. *Clinical practice and epidemiology in mental health : CP & EMH* 2006;2:17-17.

103. Yazgan IC, Dedeoglu C, Yazgan Y. Disability and post-traumatic psychopathology in Turkish elderly after a major earthquake. *International psychogeriatrics / IPA* 2006;18(1):184-7.

104. Seplaki CL, Goldman N, Weinstein M, Lin YH. Before and after the 1999 Chi-Chi earthquake: traumatic events and depressive symptoms in an older population. *Social science & medicine (1982)* 2006;62(12):3121-32.

105. Sattler DN, de Alvarado AM, de Castro NB, Male RV, Zetino AM, Vega R. El Salvador earthquakes: relationships among acute stress disorder symptoms, depression, traumatic event exposure, and resource loss. *J Trauma Stress* 2006;19(6):879-93.

106. Kisac I. Stress symptoms of survivors of the Marmara region (Turkey) earthquakes: A follow-up study. *International Journal of Stress Management* 2006;13(1):118-26.

107. Kilic C, Aydin I, Taskintuna N, Ozcurumez G, Kurt G, Eren E, et al. Predictors of psychological distress in survivors of the 1999 earthquakes in Turkey: Effects of relocation after the disaster. *Acta Psychiatrica Scandinavica* 2006;114(3):194-202.

108. Giannopoulou I, Strouthos M, Smith P, Dikaiakou A, Galanopoulou V, Yule W. Post-traumatic stress reactions of children and adolescents exposed to the Athens 1999 earthquake. *European Psychiatry* 2006;21(3):160-66.

109. Aksaray G, Kortan G, Erkaya H, Yenilmez C, Kaptanoglu C. Gender differences in psychological effect of the August 1999 earthquake in Turkey. *Nordic Journal of Psychiatry* 2006;60(5):387-91.

110. Aker AT. [1999 Marmara earthquakes: a review of epidemiologic findings and community mental health policies]. *Turk Psikiyatri Derg* 2006;17(3):204-12.

111. Acierno R, Ruggiero KJ, Kilpatrick DG, Resnick HS, Galea S. Risk and protective factors for psychopathology among older versus younger adults after the 2004 Florida hurricanes. *The American journal of geriatric psychiatry : official journal of the American Association for Geriatric Psychiatry* 2006;14(12):1051-9.

112. Roussos A, Goenjian AK, Steinberg AM, Sotiropoulou C, Kakaki M, Kabakos C, et al. Posttraumatic stress and depressive reactions among children and adolescents after the 1999 earthquake in Ano Liosia, Greece. *American Journal of Psychiatry* 2005;162(3):530-37.

113. Montazeri A, Baradaran H, Omidvari S, Azin SA, Ebadi M, Garmaroudi G, et al. Psychological distress among Bam earthquake survivors in Iran: a population-based study. *BMC public health* 2005;5:4.

114. Chang C-M, Connor KM, Lai T-J, Lee L-C, Davidson JRT. Predictors of posttraumatic outcomes following the 1999 Taiwan earthquake. *The Journal of nervous and mental disease* 2005;193(1):40-6.

115. Livanou M, Kasvikis Y, Basoglu M, Mytskidou P, Sotiropoulou V, Spanea E, et al. Earthquake-related psychological distress and associated factors 4 years after the Parnitha earthquake in Greece. *European psychiatry : the journal of the Association of European Psychiatrists* 2005;20(2):137-44.

116. Kohn R, Levav I, Garcia ID, Machuca ME, Tamashiro R. Prevalence, risk factors and aging vulnerability for psychopathology following a natural disaster in a developing country. *International Journal of Geriatric Psychiatry* 2005;20(9):835-41.

117. Kohn R, Levav I, Donaire I, Machuca M, Tamashiro R. Psychological and psychopathological reactions in Honduras following Hurricane Mitch: implications for service planning. *Revista panamericana de salud pública = Pan American journal of public health* 2005;18(4-5):287-95.

118. Altindag A, Ozen S, Sir A. One-year follow-up study of posttraumatic stress disorder among earthquake survivors in Turkey. *Comprehensive Psychiatry* 2005;46(5):328-33.

119. Desai NG, Gupta DK, Srivastava RK. Prevalence, pattern and predictors of mental health morbidity following an intermediate disaster in an urban slum in delhi : a modified cohort study. *Indian journal of psychiatry* 2004;46(1):39-51.

120. Watanabe C, Okumura J, Chiu TY, Wakai S. Social Support and Depressive Symptoms among Displaced Older Adults Following the 1999 Taiwan Earthquake. *Journal of Traumatic Stress* 2004;17(1):63-67.

121. Norris FH, Murphy AD, Baker CK, Perilla JL. Postdisaster PTSD over four waves of a panel study of Mexico's 1999 flood. *J Trauma Stress* 2004;17(4):283-92.

122. Chou FH, Chou P, Su TT, Ou-Yang WC, Chien IC, Lu MK, et al. Quality of life and related risk factors in a Taiwanese Village population 21 months after an earthquake. *The Australian and New Zealand journal of psychiatry* 2004;38(5):358-64.

123. Kuo CJ, Tang HS, Tsay CJ, Lin SK, Hu WH, Chen CC. Prevalence of psychiatric disorders among bereaved survivors of a disastrous earthquake in taiwan. *Psychiatric services (Washington, D.C.)* 2003;54(2):249-51.

124. Kolaitis G, Kotsopoulos J, Tsiantis J, Haritaki S, Rigizou F, Zacharaki L, et al. Posttraumatic stress reactions among children following the Athens earthquake of September 1999. *European Child and Adolescent Psychiatry* 2003;12(6):273-80.

125. Kilic C, Ulusoy M. Psychological effects of the November 1999 earthquake in Turkey: An epidemiological study. *Acta Psychiatrica Scandinavica* 2003;108(3):232-38.

126. Livanou M, Basoglu M, Salcioglu E, Kalendar D. Traumatic stress responses in treatment-seeking earthquake survivors in Turkey. *The Journal of nervous and mental disease* 2002;190(12):816-23.

127. Liao SC, Lee MB, Lee YJ, Weng T, Shih FY, Ma MH. Association of psychological distress with psychological factors in rescue workers within two months after a major earthquake. *Journal of the Formosan Medical Association = Taiwan yi zhi* 2002;101(3):169-76.

128. Basoglu M, SalcIoglu E, Livanou M. Traumatic stress responses in earthquake survivors in Turkey. *Journal of Traumatic Stress* 2002;15(4):269-76.

129. Najarian LM, Goenjian AK, Pelcovitz D, Mandel F, Najarian B. The effect of relocation after a natural disaster. *Journal of Traumatic Stress* 2001;14(3):511-26.

130. Maruyama S, Kwon YS, Morimoto K. Seismic intensity and mental stress after the Great Hanshin-Awaji Earthquake. *Environ Health Prev Med* 2001;6(3):165-9.

131. Goenjian AK, Molina L, Steinberg AM, Fairbanks LA, Alvarez ML, Goenjian HA, et al. Posttraumatic stress and depressive reactions among Nicaraguan adolescents after Hurricane Mitch. *American Journal of Psychiatry* 2001;158(5):788-94.

132. Shioyama A, Uemoto M, Shinfuku N, Ide H, Seki W, Mori S, et al. The mental health of school children after the Great Hanshin-Awaji Earthquake: II. Longitudinal analysis. *Seishin shinkeigaku zasshi = Psychiatria et neurologia Japonica* 2000;102(5):481-97.

133. Goenjian AK, Steinberg AM, Najarian LM, Fairbanks LA, Tashjian M, Pynoos RS. Prospective study of posttraumatic stress, anxiety, and depressive reactions after earthquake and political violence. *The American journal of psychiatry* 2000;157(6):911-6.

134. McDermott BM, Palmer LJ. Post-disaster service provision following proactive identification of children with emotional distress and depression. *The Australian and New Zealand journal of psychiatry* 1999;33(6):855-63.

135. Watson JB, Mednick SA, Huttunen M, Wang X. Prenatal teratogens and the development of adult mental illness. *Development and psychopathology* 1999;11(3):457-66.

136. Sharan P, Chaudhary G, Kavathekar SA, Saxena S. Preliminary report of psychiatric disorders in survivors of a severe earthquake. *American Journal of Psychiatry* 1996;153(4):556-58.

137. Najarian LM, Goenjian AK, Pelcovitz D, Mandel F, Najarian B. Relocation after a disaster: Posttraumatic stress disorder in armenia after the earthquake. *Journal of the American Academy of Child and Adolescent Psychiatry* 1996;35(3):374-83.

138. Kato H, Asukai N, Miyake Y, Minakawa K, Nishiyama A. Post-traumatic symptoms among younger and elderly evacuees in the early stages following the 1995 Hanshin-Awaji earthquake in Japan. *Acta Psychiatrica Scandinavica* 1996;93(6):477-81.

139. David D, Mellman TA, Mendoza LM, Kulick-Bell R, Ironson G, Schneiderman N. Psychiatric morbidity following Hurricane Andrew. *Journal of Traumatic Stress* 1996;9(3):607-12.

140. Karanci AN, Rustemli A. Psychological consequences of the 1992 Erzincan (Turkey) earthquake. *Disasters* 1995;19(1):8-18.

141. Goenjian AK, Pynoos RS, Steinberg AM, Najarian LM, Asarnow JR, Karayan I, et al. Psychiatric comorbidity in children after the 1988 earthquake in Armenia. *Journal of the American Academy of Child and Adolescent Psychiatry* 1995;34(9):1174-84.

142. Hardin SB, Weinrich M, Weinrich S, Hardin TL, Garrison C. Psychological distress of adolescents exposed to Hurricane Hugo. *Journal of Traumatic Stress* 1994;7(3):427-40.

143. Thompson MP, Norris FH, Hanacek B. Age differences in the psychological consequences of Hurricane Hugo. *Psychol Aging* 1993;8(4):606-16.

144. Jeney-Gammon P, Daugherty TK, Finch AJ, Jr., Belter RW, Foster KY. Children's coping styles and report of depressive symptoms following a natural disaster. *The Journal of genetic psychology* 1993;154(2):259-67.

145. Lima BR, Chavez H, Samaniego N, Pai S. [Psychiatric disorders among victims of disasters in Ecuador]. *Boletin de la Oficina Sanitaria Panamericana. Pan American Sanitary Bureau* 1992;113(1):28-34.

146. Shore JH, Tatum EL, Vollmer WM. Evaluation of mental effects of disaster, Mount St. Helens eruption. *Am J Public Health* 1986;76(3 Suppl):76-83.

147. Murphy SA. Stress levels and health status of victims of a natural disaster. *Research in nursing & health* 1984;7(3):205-15.

148. Grecu G, Csiky K, Munteanu I. [Study of a group of 87 patients with depressive states triggered by the floods of May 1970]. *Neurologia, psihiatria, neurochirurgia* 1972;17(2):109-16.
